# Antisocial Personality Traits, Substance Use, and Somatization: A Brief Consideration of Their Interrelation

**DOI:** 10.3390/ijerph21010061

**Published:** 2024-01-03

**Authors:** Eamonn Arble

**Affiliations:** Department of Psychology, Eastern Michigan University, Ypsilanti, MI 48197, USA; earble2@emich.edu; Tel.: +734-487-5988

**Keywords:** antisocial, psychopathy, alcohol, somatization

## Abstract

The relationship between antisocial personality traits and the expression of somatic symptoms has been the subject of several theoretical and empirical investigations. The present study sought to advance the understanding of the relationship between these variables by testing two moderation models. It was hypothesized that the relationship between antisocial traits and somatization would be moderated by alcohol use, such that the presence of alcohol dependence would strengthen the relationship between antisocial traits and somatization. It was also hypothesized that gender would play a moderating role in the relationship between ASPD and somatization, such that the relationship would be stronger among women than among men. These models were tested in a sample of 787 criminal offenders. Gender did not emerge as a significant moderator in the relationship between antisocial traits and somatization. Although substance use did significantly moderate the relationship between antisocial traits and somatization, the direction of the effect ran counter to expectations: among participants reporting a history of alcohol dependency, the relationship between antisocial features and somatization was diminished. The implications of these findings are discussed.

## 1. Introduction

Comorbidity among psychological diagnostic categories has been a longstanding source of intrigue and frustration for clinicians and researchers alike [1]. This issue is particularly pronounced among personality disorders, which demonstrate significant conceptual and empirical overlap, not only with one another, but also with a range of other forms of psychopathology [2]. These comorbidities can make the treatment and assessment of individual patients more difficult and can further make extrapolation from individual research findings more tenuous. Conversely, these interrelations offer opportunities for unique investigations to identify cross-cutting factors of particular importance in case conceptualization and treatment, bringing to light to new ways of understanding existing personality constructs. Within this framework, the present investigation explores one of the more historically intriguing relationships found between antisocial personality disorder (ASPD) and somatic symptom disorder.

As defined by the *Diagnostic and Statistical Manual of Mental Disorders* (5th ed., text rev.; DSM-5-TR [3]), the hallmark feature of ASPD is a consistent pattern of behavior in which the rights and concerns of others are disregarded and violated. Individuals diagnosed with ASPD are characterized by the violation of laws/rules, deceitfulness, aggression, impulsivity, and several other symptoms reflective of a general recklessness. As with any personality disorder, the described patterns must be pervasive; temporary bouts of impulsivity and self-centeredness do not qualify for the diagnosis and are generally indicative of a more transient issue. In the case of ASPD, this pervasiveness must also extend into childhood. Although ASPD cannot be diagnosed until the age of 18, to meet DSM-5-TR diagnostic criteria, the individual must also have evidence of Conduct Disorder before the age of 15. In other words, the diagnosis reflects a pattern of disruptive and malicious behavior that begins in childhood or adolescence and continues into adulthood.

ASPD is one of the most controversial and clinically significant personality disorders. Individuals diagnosed with ASPD are more likely to engage in destructive behavior [4], be incarcerated [5], and experience poorer outcomes relating to mental and physical health [6]. It is comorbid with a number of psychiatric diagnoses and overall disruptions to quality of life [7]. Despite this clinical relevance, the diagnosis has been hampered by questionable practical utility and accompanying debate about its ultimate nature [8]. ASPD is a behaviorally focused diagnosis within the DSM, but the DSM definition has been criticized for neglecting the affective and interpersonal traits that may be core to related, and potentially more useful, constructs such as psychopathy [9]. Indeed, recent investigations have found strong empirical support for the conceptualization of the pathology in terms of related but distinct dimensional characteristics, of which the externalizing behaviors emphasized in the DSM are only one component [10].

Somatic symptom disorder, as defined by the DSM-5-TR, is characterized by the presence of two features: (1) the existence of somatic symptoms that are a source of distress or practical disruption and (2) an excessive or atypical response to the presence of the somatic symptoms. Regarding the latter, the reactions are defined by a disproportionate or abnormal preoccupation with the symptoms, often resulting in anxious and ruminative focus on the nature and implications of their presence. Previous conceptualizations of the diagnosis focused on the lack of medical explanation for the individual’s symptoms, whereas the modern incarnation emphasizes the pathological interpretation of the symptoms [11]. The nature of the symptoms is highly variable, with some individuals experiencing pain in highly localized areas (e.g., chest pain) and others experiencing symptoms in a more general sense (e.g., fatigue) [12].

At first glance, these disorders bear little resemblance to one another. ASPD is a disorder of externalization and rule violation, while somatic symptom disorder is a disorder of anxious preoccupation. However, the link between the two diagnoses is relatively well established [13]. Somatic symptoms are a common symptom among individuals seeking or referred for psychological treatment, and this relationship is seen in medical, outpatient, and inpatient settings [14]. A meta-analytic investigation by Bornstein and Gold [15] found that somatic symptom disorder comorbidities with personality disorders, including ASPD, may be generally classified as having a medium effect size, suggesting a noteworthy connection. This same meta-analytic investigation identified that both pathologies may have a shared etiology in family disturbances and anxious attachment styles, an assertion bolstered by the evidence of covariance of ASPD and somatic symptoms among individuals and their families [16].

It has been further noted that the two diagnoses may share certain biological underpinnings. Both disorders present with low serotonin levels, leading some researchers to posit that genetic markers associated with decreased serotonin may offer the source of a shared etiology [17]. Espiridion and Kerbel [17] cite the dopa decarboxylase (*DDC*) gene as a likely target, noting that the gene and its various polymorphisms may play a significant role in various serotonergic pathways. Most importantly, the *DDC* gene minor allele rs11575542 has been associated with both somatic symptoms as well as marked increase in sensation-seeking behavior, offering a promising explanation for the apparent genetic link between the two conditions. 

In a seminal article on the topic, Lilienfeld [18] identified several conceptual models to explain the relationship between ASPD and somatic symptoms. One identified model, the negative emotionality model (NE) [19], is of particular importance. This model describes a process in which the reckless and impulsive life choices of the antisocial individual result in an elevated experience of negative emotions. For example, an individual who is deceitful and aggressive may soon find themselves socially isolated and facing severe economic and legal consequences; these consequences then give rise to negative emotions (e.g., sadness and anxiety). A combination of physical and internal injuries as a result of impulsive behavior, as well as a discomfort within recognizing or processing one’s own emotions, may ultimately translate to the individual experiencing somaticized symptoms. Lilienfeld and Hess [20] would later extend this work by identifying the mediating role of negative emotions in the relationship between psychopathic personality traits and somatization.

Lilienfeld [18] also highlights another intriguing aspect of the relationship between the disorders, namely, the possibility of somatization as a sex-differentiated expression of ASPD. ASPD is diagnosed with significantly greater frequency among men than women [21]. While this might reflect a biological disposition, an alternative hypothesis is that women may express their antisocial traits in a gender-specific fashion. The diagnostic criteria of ASPD, with its focus on overt and aggressive behaviors, could neglect other manifestations of the pathology, namely somatic symptoms. Somatic symptom disorder is diagnosed with greater frequency among women than men [21] and might represent a pathway for women to exhibit a kind of indirect relational aggression that represents the same underlying antisocial traits. Unfortunately, the role of psychopathy among women has been comparatively understudied. Nonetheless, there is evidence to suggest that gender may interact with the expression and comorbidities of antisocial traits [22,23]. And there is some research to suggest that among women specifically, there may be a relationship between psychopathic traits and somatization [24], but other work has failed to replicate this finding [25].

This line of research carries several key implications. First, the relationship between antisocial characteristics and somatic symptoms is complex and likely acted upon by a number of sources. Second, within the framework of the NE model, poor decision making resulting in adverse consequences is the key component of emotional distress; thus, behaviors that increase the likelihood of negative outcomes will be particularly relevant for understanding subsequent somatization. And finally, behaviors that result in health problems or atypical physical sensations may be particularly likely to be experienced as a form of somatization. In totality, these implications suggest the potential importance of a factor that has demonstrated significant relationships with both ASPD and somatic symptom disorder: substance use. 

The relationship between ASPD and substance use has been thoroughly researched [26]. When considering alcohol use, the numbers are truly dramatic. In one study, it was found that approximately 75% of individuals with ASPD also met criteria for alcohol use disorder (AUD) [27]. Other estimates suggest an AUD prevalence of 68% among those diagnosed with ASPD [28]. The strong overlap between the disorders may be the result of many factors. Traumatic childhood experiences, peer selection, and attachment difficulties are common to both disorders [29]. Other research has noted that impulsivity or behavioral inhibition are both common to, and potentially explain the relationship between, AUD and ASPD [30]. Genetic variance associated with the category of behavioral under-control appears to account for a significant amount of the variance within the two disorders [31], leading many to conclude that both should be conceptualized under the larger heading of externalizing disorders [32].

The combination of antisocial traits and substance use is both frequent and deleterious [33]. The disinhibition and recklessness of ASPD may naturally facilitate greater alcohol consumption [34]. Furthermore, alcohol consumption may produce increased disinhibition, leading to more frequent and severe antisocial behavior [35]. As articulated by the NE model, increased alcohol consumption could produce additional negative consequences for an individual, thereby producing more negative emotions. In the aforementioned example of an individual behaving in a deceitful and aggressive manner, these destructive behaviors may be amplified with the addition of alcohol, perhaps creating an even greater degree of resulting negative emotion. 

In addition to greater negative emotions in a general sense, it may also be the case that such a pattern of ASPD and substance use is uniquely suited for the creation of somaticized symptoms [36]. First, alcohol use can create a number of physical symptoms that can serve as a source of distress [37]. Headaches, abdominal pains, and fatigue are only some of the symptoms produced by alcohol use and withdrawal that might ultimately become the source of abnormal preoccupation, the very requirement of somatic symptom disorder. Second, the use of alcohol may not only be a sign of impulsiveness but may also reflect an effort at coping with emotional distress.

Psychopathic traits tend to be highly correlated with the use of maladaptive coping strategies such as avoidant coping [38]. Avoidant coping is defined as an avoidance of distressing emotions or problems via distraction. Although avoidant coping can be effective when combined with more active strategies, it can be harmful when used solely or excessively [39]. At best, avoidant coping can only provide a brief respite, as it does not address the individual’s internal needs or external demands. Alcohol use can be a potent form of avoidance, providing both distraction and emotional numbing, though its use comes at a grave cost [40]. An individual using alcohol to blunt their feelings or avoid thinking about their problems may be decreasing their health, increasing their likelihood of making bad decisions, and almost certainly will not improve their circumstances. Nonetheless, despite this maladaptive quality, alcohol use in this context could be conceptualized as a form of coping.

The use of alcohol as a form of coping has been demonstrated repeatedly [41]. Furthermore, the link between alcohol use and somatization, including as a response to stressful experiences, has been found in large community samples [42]. Most intriguingly though, there is evidence that among individuals with high levels of antisocial traits, substance use has been linked to increased somatization [43]. This connection suggests that among individuals high on antisocial traits, alcohol use may not only represent an act of impulsivity but may also reflect an effort to cope with painful thoughts and emotions, the distress of which may ultimately manifest in the form of somatic symptoms.

The interrelationship between these variables clearly merits further study. The present study sought to elucidate the matter through the testing of a moderation model including antisocial traits, somaticized symptoms, and alcohol use. It was hypothesized that:

1. The relationship between antisocial traits and somatization is moderated by alcohol use, such that the presence of alcohol dependence will strengthen the relationship between antisocial traits and somatization.

To examine the theory of somatization as a sex-differentiated expression of ASPD, it was also hypothesized that:

2. Gender would play a moderating role in the relationship between ASPD and somatization, such that the relationship would be stronger among women than among men.

## 2. Materials and Methods

### 2.1. Participants

Data were taken from the Pathways to Desistance Study, a multisite study that followed serious juvenile offenders from adolescence to young adulthood [44]. Participants were initially recruited for the study if they had committed a serious offense (e.g., a felony) and were adjudicated either delinquent or found guilty by the court systems of Philadelphia, Pennsylvania, or Phoenix, Arizona. Participants in the study completed a baseline interview assessment, followed by subsequent interviews at six-month intervals over a three-year period. Additional interviews were conducted following the participant’s release from a residential facility. Additional study details are reported in Schubert et al. [45].

This longitudinal study included follow-up at 72 months, at which time the Personality Assessment Inventory (PAI) [46] was added to the study protocol. The dataset included 787 respondents with complete data on the variables of interest (from the original sample of 1354 respondents) at the 72-month time point. The sample included young adults, age 20–24 years (*M* = 22.03, *SD* = 1.14) and by majority self-reported identifying as male (*n* = 661; 84%). The sample was racially and ethnically diverse: 186 (23.6%) reported as non-Hispanic white, 316 (40.2%) as Black, 246 (31.3%) as Hispanic, and 39 (5%) as other. 

### 2.2. Measures

Personality Assessment Inventory (PAI) [46]: The PAI is a 344-item omnibus self-report measure of psychological functioning. Items are scored from 1 (*Not at all, False*) to 4 (*Very True*). Its reliability and validity have been demonstrated in numerous studies [47,48] (from the publicly shared dataset, only summary scores were distributed, and at the time of this writing, the internal reliability of the scale in this sample has not been published). The present study utilized the results from the antisocial scale, consisting of its three subscales: egocentricity, stimulus seeking, and antisocial behaviors. The egocentricity subscale focuses on the assessment of callousness, lack of empathy, and disregard for the welfare of others. The stimulus-seeking subscale focuses on risk taking in pursuit of novel experiences, excitement seeking, and disregard of potential consequences. The antisocial behaviors subscale focuses on conflict with authority figures, rule-breaking behavior, and the violation of social norms. 

Brief Symptom Inventory (BSI) [49]: The BSI is a 53-item self-report measure of a range of clinical symptoms. Items are scored from 0 (*Not at all*) to 4 (*Extremely*). Its reliability and validity have been demonstrated in numerous studies [49,50]. High reliability has been previously reported for this sample for the somatization (Cronbach’s α = 0.80) and global severity (Cronbach’s α = 0.96) scales [51]. The somatization subscale focuses on the experience of symptoms that are somatic in nature, for example, dizziness. The Global Severity Index captures the respondent’s overall level of pathology across all scales of the BSI.

Composite International Diagnostic Interview (CIDI) [52]: The CIDI is a structured diagnostic interview meant to assess a wide range of clinical issues. When properly administered, the instrument appears to be an effective diagnostic tool [53]. The present study utilized the results from the alcohol dependence module. This module contains questions about the diagnostic criteria for alcohol dependence, including the experience of alcohol-related symptoms and consequences. 

### 2.3. Statistical Analysis

All analysis was completed in SPSS software (v. 28.0) [54]. Prior to hypothesis testing, descriptive statistics and comparisons by gender were generated with measures of central tendency, frequency, and independent sample *t*-test (adjustments were made as needed when the assumption of equality of variances was not met between groups). Hypotheses were tested in univariate general linear models including cases with complete data on the variables of interest (*n* = 787). Approximately 42% of the sample had data missing not at random (Little’s χ^2^ (9) = 46.23, *p* < 0.001), and in most cases, the data were missing due to incomplete assessments across multiple measures, which limits options to estimate and replace missing data. Given the large sample size, listwise deletion was chosen for model estimation. Assumptions of multivariate normality and homoscedasticity of the residuals, homogeneity of variance between groups, and statistical leverage were assessed in the model following standard procedures. Robust multivariate tests are reported for all hypothesis tests. Models were tested with alcohol dependency and gender as 2-level factors; raw scores of PAI antisocial behavior, egocentricity, and stimulus seeking were included as continuous predictors. Interactions between PAI subscales and alcohol dependency were tested and removed from the model if not significant (α = 0.05). Complex effects were decomposed with graphing and bivariate correlations stratified by alcohol dependency. BSI somatization raw score was tested as an outcome of interest, and a second model predicting BSI global severity raw score was tested as a hypothesized negative control comparison. Analyses are reported for the entire available sample and were repeated, excluding cases with a statistically high leverage indexed by Cook’s distance to confirm the pattern of results. The available sample with complete data (*n* = 787) provided 80–95% power to detect small omnibus effects (*f*^2^ = 0.02–0.03) to significance [55].

## 3. Results

### 3.1. Descriptive Analysis

The sample was characterized with multiple measures to evaluate alcohol dependency, somatization and anti-social attributes. Participants reported the frequency and severity of drinking and were classified for clinical alcohol dependency at any time during the study: 96 (12.2%) met the criteria for alcohol dependency, and 691 (87.8%) did not. Analyses of PAI anti-social attributes and BSI reported symptoms were tested with raw scores. BSI somatization ranged from 0 to 3.0 (*M* = 0.26, *SD* = 0.46) and GSI ranged from 0.02 to 3.13 (*M* = 0.39, *SD* = 0.46). Converting scale scores to standardized *t*-scores, only *n* = 26 (3.3%) had clinical elevations in somatization and *n* = 22 (2.8%) clinically elevated in GSI; therefore, the analyses largely considered sub-clinical elevations in the reported symptoms. PAI anti-social behaviors ranged from 1 to 24 (*M* = 12.31, *SD* = 3.99), egocentricity ranged from 0 to 21 (*M* = 4.17, *SD* = 3.61), and stimulus seeking ranged from 0 to 24 (*M* = 7.95, *SD* = 4.51). Converting the total scale score to standardized values, the *t*-scores ranged from 40 to 108 (*M* = 62.38, *SD* = 11.36); *n* = 187 had clinical elevations above *t-*score = 70. As expected, female participants reported average higher BSI somatization (*M* = 0.40, *SD* = 0.57) than men (*M* = 0.24, *SD* = 0.43; *t* = −3.08, *p* = 0.002). Male participants reported elevations across all PAI antisocial subscales as compared to female participants (all *t* ≥ 2.58, *p*’s < 0.01). Unstandardized scale scores were included for further analysis; see Table 1 for bivariate correlations among study variables.

### 3.2. Moderation Model: Alcohol Dependency Moderates the Relation between Anti-Social Stimulus Seeking and Somatization

**Figure 1:**
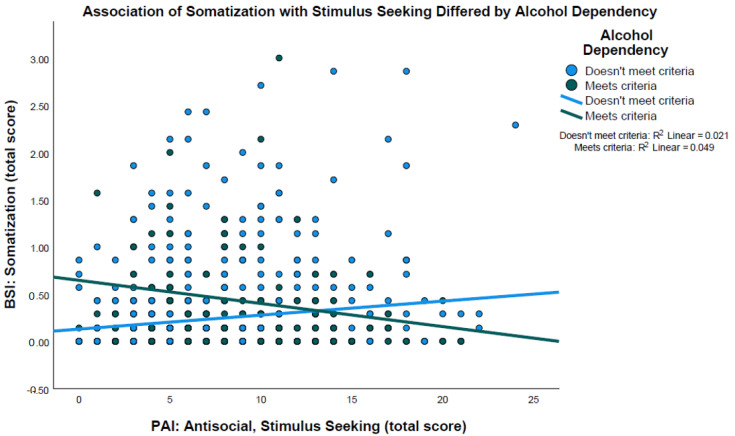
The association between antisocial stimulus-seeking behavior and reported somatization was moderated by alcohol dependency [(F (1, 780) = 13.93, *p* < 0.001; η_p_^2^ = 0.02]. Among young adults reporting no alcohol dependency, the association was positive (r = 0.15, *p* < 0.001), whereas this effect was negative in those reporting alcohol dependency (r = −0.22, *p* < 0.001).

In a univariate general linear model, PAI subscales were entered as predictors of BSI somatization, including alcohol dependency and its interaction with the anti-social subscales. The interactions of alcohol dependency with antisocial behaviors [F (1, 775) = 2.97, *p* = 0.09] and egocentricity [F (1, 775) = 0.96, *p* = 0.33] were not statistically significant and were removed from further model testing. All interactions of the PAI subscales with gender were also not significant [all F (1, 775) = 0.81–2.26, *p*’s ≥ 0.13] and removed. In the reduced model, the effect of stimulus seeking on somatization was moderated by alcohol dependency [F (1, 780) = 13.93, *p* < 0.001; η_p_^2^ = 0.02]. This interaction was supported, accounting for the unique effect of alcohol dependency [F (1, 780) = 19.40, *p* < 0.001; η_p_^2^ = 0.02] and gender [F (1, 780) = 13.48, *p* < 0.001; η_p_^2^ = 0.02]. The moderated effect indicated a positive correlation between stimulus seeking and somatization among young adults reporting no alcohol dependency (r = 0.15, *p* < 0.001), whereas this effect was negative in those reporting alcohol dependency (r = −0.22, *p* < 0.001; Figure 1).

Taking into account the complex effect of alcohol dependency, the unique effects of stimulus seeking [F (1, 780) = 3.54, *p* = 0.06], antisocial behavior [F (1, 780) = 3.44, *p* = 0.06], and egocentricity [F (1, 780) = 3.26, *p* = 0.07] were not significant. When repeating the analysis but excluding cases that introduced a statistical leverage based on Cook’s distance of the model residuals (*n* = 47), all results were comparable, including the moderated effect (F = 14.35, *p* < 0.001). In addition, the unique effects of antisocial behaviors (F = 8.78, *p* = 0.003) and stimulant seeking (F = 10.60, *p* = 0.001) were in the same direction and reached statistical significance. However, following best practices [56], analysis of the complete sample is interpreted to prioritize external validity having confirmed the substantive conclusion of stimulus-seeking effects moderated by alcohol dependency after removing leverage cases.

To rule out the possibility of a general elevation in psychological symptoms, a similar model was tested with BSI global symptom severity. In this model, there was no evidence of alcohol dependency moderating the effect of PAI subscales [all F (1, 776) = 0.83–2.73, *p* ≥ 0.10], nor any interactions with gender [all F (1, 776) = 0.28–3.10, *p* ≥ 0.08]. After removing the interaction terms that were not statistically significant, alcohol dependency [F (1, 782) = 8.58, *p* = 0.003; η_p_^2^ = 0.01] and female gender [F (1, 782) = 6.95, *p* = 0.01; η_p_^2^ = 0.01] were associated with higher symptom severity. Elevations in antisocial behaviors [F (1, 782) = 13.15, *p* < 0.001; η_p_^2^ = 0.02] and egocentricity [F (1, 782) = 4.68, *p* = 0.03; η_p_^2^ = 0.01] were associated with greater reported symptom severity, but stimulant seeking was not uniquely associated with it [F (1, 782) = 1.33, *p* = 0.25].

## 4. Discussion

The present study sought to explore the association between antisocial traits and somatic symptoms, with the hypothesis that alcohol dependence would moderate the relationship between the two. Although a moderated effect was identified, the hypothesis that the presence of alcohol dependence would strengthen the relationship between antisocial traits and somatic symptoms was not supported. Conversely, the opposite pattern was identified; among participants reporting a history of alcohol dependency, the relationship between antisocial features and somatization was diminished.

It was hypothesized that alcohol dependence would strengthen the relations between antisocial traits and somatization for several reasons. First, if alcohol use reflects emotional distress, it seems reasonable to suggest that increased alcohol use could be reflective of greater levels of emotional pain. As noted in the NE model, the presence of this distress could translate to a need for greater somatization. Second, alcohol use itself might create additional negative emotions through an increase in destructive behaviors. In other words, individuals using alcohol excessively might make costly mistakes with greater frequency, thereby creating greater negative emotions in their lives. Again, in accordance with NE theory, this could produce a greater likelihood of somatization. Finally, alcohol consumption can create a host of physical symptoms that can ultimately become a source of anxious preoccupation as required by somatic symptom disorder [57]. That the present results do not support these lines of argument raises interesting questions about the role of alcohol use within individuals with traits of ASPD.

One potential explanation for this finding may be strongly related to a primary limitation of the present research. Although the sample was drawn from a group of juvenile offenders, and the PAI antisocial scales are valid measures of the construct, there is an important distinction between the presence of antisocial traits and a diagnosis of ASPD [58]. This is complicated by the fact that even the diagnostic labels themselves are insufficient [59]. A diagnosis of ASPD is, by design, a behaviorally anchored list of symptoms that can be clinically assessed and diagnosed. This diagnosis, while useful, is often criticized as an insufficient reflection of the more clinically relevant construct of psychopathy [60]. Although the present research is derived from a relevant sample, it is not dealing with clinical constructs per se, but rather, traits that are of relevance to the constructs. As it has become increasingly clear that the construct of psychopathy requires a multifaceted or dimensional conceptualization [61], it might also be the case that the present results are only reflective of a small piece of the broader puzzle of antisocial traits and somatization. Indeed, using a prominent two-factor model of psychopathy distinguishing between “primary” and “secondary” psychopathy [62], researchers have found significant differences in these subtypes on outcomes including symptom profiles, coping strategies, and adaptive functioning [20,63]. A more nuanced assessment of the clinical constructs within the study, including a distinction between individuals crossing clinical thresholds and those with more normative trait profiles, could provide different results.

Nonetheless, the proposed relationship between alcohol dependence and negative emotions functioned differently than expected. Although alcohol use may indicate emotional distress, within this sample, it may also be more accurately conceptualized as an extension of stimulus seeking. This finding may reflect the limitation noted above that the present analysis is driven by an aspect of ASPD (i.e., stimulus seeking) among a sample of offenders, as opposed to ASPD or psychopathy directly. In might also be the case that, rather than alcohol use serving as a vehicle to translate emotional distress into physical symptoms, alcohol use among this sample instead reflected a maladaptive coping strategy that prevented the need for somatic symptoms. In a curious way, individuals engaged in excessive alcohol use may have had an avenue to engage with their feelings of restlessness or negative emotions outside of manifesting physical symptoms. For those who did not have this outlet, pathological though it may be, somatization may have been the natural outcome. This may also reflect that alcohol usage in a relatively younger sample likely does not carry the same implications as among older adults [64]. Greater social acceptance of excessive drinking, combined with more physical resilience in the face of physical symptoms from alcohol consumption, could render the relationship between drinking and somatization fundamentally different in this young adult sample.

The present study also investigated the role of gender, both as a moderator of the association between ASPD and somatization and as a control in the proposed alcohol moderation model. In both cases, the role of gender was not significant. Some have argued that the ASPD diagnostic criteria are geared towards overt acts of hostility and criminality that are more likely to be engaged in by men, neglecting the expressions of the pathology that women may engage in more frequently [65]. In this understanding, features of somatic symptoms may reflect a sex-specific phenotypical expression of ASPD. Consistent with population estimates, in the present sample, women scored higher in somatization and lower on antisocial scales. However, gender did not emerge as a moderating variable in the relationship between ASPD and somatization. The proposed moderating role of gender in this relationship has not been universally observed, so in some respects, this finding is not entirely surprising [66].

The failure of gender to emerge as a significant moderator requires two important caveats. First, women were significantly outnumbered by men in the sample. It is possible that with a larger sample of female offenders, the results would have been different. Second, in the argument that women express psychopathic features via somatization, it is noted that traditional measures of ASPD symptoms may be overlooking gender-specific manifestations of the disorder. Measures that capture the underlying features of ASPD, or perhaps its genetic markers, may provide a more accurate test of the role of gender in the relationship between psychopathic traits and somatization. Although the present results suggest that even among a sample of offenders, women are more likely to report somatization and less likely to report symptoms of ASPD, it does not appear that somatization is a gender-specific manifestation of underlying ASPD traits.

As noted previously, the assessments of the included clinical constructs were limited. Fuller assessments, including a multimethod assessment approach, could provide important additional information. This limitation is further exacerbated by the finding that data were not missing at random over the course of the study. The use of listwise deletion may have provided some protection against the implications of the missing data, but there remains the possibility that traits of relevance to the present investigation were differentially affected as participants declined participation. As such, all of the present conclusions should be interpreted with caution.

## 5. Conclusions

The relationship between antisocial personality traits, substance use, and somatization proved to be quite complex. Alcohol dependence did serve as a moderator in the relationship between the PAI antisocial subscale of stimulus seeking and the BSI scale of somatization, even after controlling for the effects of gender and general pathology. However, the direction of the relationship ran counter to expectations. The moderated effect indicated a positive correlation between stimulus seeking and somatization among young adults reporting no alcohol dependency, whereas this effect was negative in those reporting alcohol dependency. This finding has important implications for the field’s understanding of the interaction between these constructs. First, heterogenous clinical constructs may give rise to heterogenous methods of emotional expression and regulation. For some individuals, the consumption of alcohol may represent a state of distress; for others, it could represent an impulse to engage in celebratory activities. Similarly, engaging in reckless behavior could reflect a means of coping with emotional pain, or it could reflect an impulsive character. Because of this ambiguity, it is important that researchers and clinicians explore not only the correlation between clinical features, but of equal importance, the meaning and function underlying an individual’s behavior. The “why” of the behavior may provide critical insight not captured by the application of a diagnostic label. Second, and highly related to the first, thorough assessment remains an essential aspect of clinical practice and research. Particularly in the realm of personality disorders, the importance of dimensional models is being increasingly recognized [67]. These models seek to provide a more nuanced view of intradiagnostic factors that operate within the diagnostic categories, helping to provide a language to describe the variation occurring across individuals. For those with a diagnosis of ASPD, the role of somatic symptoms as an expression of emotional distress should be explored. However, the present results suggest that stimulus seeking may be the most relevant aspect of ASPD for understanding the connection to somatic symptoms, such that individuals with antisocial features, particularly those with prevalence experiences of stimulus seeking, should be evaluated for both substance use and the presence of somatic symptoms. Third, the presence of ASPD, either as a diagnosis or in the form of antisocial traits, should not be taken as evidence of a lack of distress. Features of ASPD are often accompanied by emotional pain [68], and the present results suggest this pain can be expressed in many different ways that could mistakenly be overlooked by the individuals themselves, by treating clinicians, or by researchers.

An additional important finding is that gender did not emerge as a moderator in the relationship between antisocial traits and somatization, though as expected, women reported higher levels of somatization and lower levels of ASPD than men. With the limitations of the sample size (i.e., fewer female participants) and the narrow assessment approach in mind, the present results nonetheless do not provide evidence of a sex-specific ASPD profile. Although women undoubtedly experience societal expectations that may affect the specific manifestation of antisocial features, there is no compelling evidence that somatic symptom disorder should be characterized as a female version of ASPD. Although women may be more likely to express somatic symptoms than men, it might also be the case the researchers are inclined to focus on somatization in women, while neglecting the more antisocial facets [69]. The field should be cautioned against this, as there appears to be no reason to conclude that women necessarily engage in more indirect forms of antisocial behavior.

## Figures and Tables

**Table 1:** Descriptive statistics and bivariate correlations among study variables.

			Variable				
	Variable	*M* (*SD*)	1	2	3	4	5
1.	BSI Somatization	0.26 (0.46)	1.00				
2.	BSI Global Severity	0.39 (0.46)	0.77	1.00			
3.	PAI Antisocial Behavior	12.31 (3.99)	0.13	0.25	1.00		
4.	PAI Egocentricity	4.17 (3.61)	0.14	0.23	0.53	1.00	
5.	PAI Stimulus Seeking	7.95 (4.51)	0.10	0.22	0.59	0.66	1.00

Note: Descriptive statistics of the mean (*M*) and standard deviation (*SD*) and Pearson correlation coefficients are reported for unstandardized scale scores that are included in further analysis. All correlations were statistically significant, *p* < 0.01.

## Data Availability

The data presented in this study are openly available in the Inter-university Consortium for Political and Social Research [distributor], 14 March 2016. https://doi.org/10.3886/ICPSR29961.v2.
